# Trends in police complaints and arrests on New York City subways, 2018 to 2023: an interrupted time-series analysis

**DOI:** 10.1186/s40621-024-00501-9

**Published:** 2024-04-26

**Authors:** Leah E. Roberts, Christina A. Mehranbod, Brady Bushover, Ariana N. Gobaud, Evan L. Eschliman, Carolyn Fish, Siddhesh Zadey, Xiang Gao, Christopher N. Morrison

**Affiliations:** 1 Department of Epidemiology, Mailman School of Public Health, Columbia University, 722 W 168th St, Rm 505, New York, NY 10032 USA; 2 Department of Epidemiology and Preventive Medicine, School of Public Health and Preventive Medicine, Monash University, Melbourne, VIC 3004 Australia

**Keywords:** Crime, Transit, Assault, Time series, New York City

## Abstract

**Background:**

Public transportation use is influenced by perceptions of safety. Concerns related to crime on New York City (NYC) transit have risen following NYC’s COVID-19 pandemic state of emergency declaration in 2020, leading to declines in subway ridership. In response, the most recent mayoral administration implemented a Subway Safety Plan in 2022. This study aimed to quantify the effects of the COVID-19 pandemic and the Subway Safety Plan on rates of complaints to and arrests by the New York City Police Department (NYPD) Transit Bureau.

**Methods:**

Using publicly available data on complaints and arrests, we conducted interrupted time-series analyses using autoregressive integrated moving average models applied to monthly data for the period from September 2018 to August 2023. We estimated changes in the rates of complaints to and arrests by the NYPD Transit Bureau before and after: (1) the COVID-19 pandemic state of emergency declaration (i.e., March 2020), and (2) the announcement of the Subway Safety Plan (i.e., February 2022). We also examined trends by complaint and arrest type as well as changes in proportion of arrests by demographic and geographic groups.

**Results:**

After the COVID-19 pandemic declaration, there was an 84% increase (i.e., an absolute increase of 6.07 per 1,000,000 riders, CI 1.42, 10.71) in complaints to the NYPD Transit Bureau, including a 99% increase (0.91 per 1,000,000 riders, CI 0.42, 1.41) in complaints for assault and a 125% increase in complaints for harassment (0.94 per 1,000,000 riders, CI 0.29, 1.60). Following the Subway Safety Plan there was an increase in the rate of arrests for harassment (0.004 per 1,000,000 riders, CI 0.001, 0.007), as well as decreases in the proportion of arrests for individuals racialized as White (− 0.02, CI − 0.04, − 0.01) and proportion of arrests in the borough of Manhattan (− 0.13, CI − 0.17, − 0.09).

**Conclusions:**

The increased rates of complaints to the NYPD Transit Bureau following the onset of the COVID-19 pandemic remained elevated following the enactment of the Subway Safety Plan. Further evaluation efforts can help identify effective means of promoting safety on public transportation.

**Supplementary Information:**

The online version contains supplementary material available at 10.1186/s40621-024-00501-9.

## Background

Public transportation has been shown to have myriad health benefits for individuals, communities, and the environment, including reductions in traffic crashes and air pollution and improvements to physical health (Public Transportation in the US: A Driver of Health and Equity | Health Affairs Brief 2024; Xiao et al. 2019). Some demographic groups in the United States (US) depend more on public transit than others, including individuals who are younger, lower income, or racialized as Black or Hispanic (Bureau UC. Census.gov 2019), and inequities in access to public transportation can exacerbate socioeconomic inequality (Kaufman et al. 2014). One major threat to equity in transit access is perceived safety. Transit stations and trains can be areas associated with violence, as they provide opportunities for violent interactions between individuals who have ample means of escape after causing harm and individuals who may be preoccupied or distracted on their transit journeys (Li and Kim 2023; Wu and Ridgeway 2021). After the COVID-19 pandemic began, concerns regarding increased risk of infectious disease, increased reports of crime, and decreased perceptions of safety have contributed to sharp declines in public transit ridership globally (Qi et al. 2023; Bosman et al. 2022; Böcker et al. 2023).

Concerns about decreased ridership and perceptions of safety on transit are particularly salient in New York City (NYC). The NYC subway is a vital part of transportation infrastructure, operating 24 h a day, 365 days a year, and connecting 665 miles and millions of New Yorkers (MTA 2024). There has been widespread media coverage of violent attacks within the subway system (Zraick et al. 2022; New York City Choking Death Revives Debate Over Subway Crime | Reuters 2024; New York Subway Crime: What is Perception, What is Real, and How to Fix it | CNN 2024), as well as increased fear among transit riders; according to a Metropolitan Transportation Authority (MTA) survey from the Spring of 2022, approximately 60% of individuals who reported using the subway less frequently indicated concern for personal security as a contributing factor (MTA 2022).

In February 2022, NYC Mayor Eric Adams announced the Subway Safety Plan, which had stated aims of addressing concerns about safety as well as supporting those experiencing homelessness and serious mental illness (The Official Website of the City of New York 2022). The plan included deployment of multi-disciplinary teams of homeless service and mental health professionals as well as increased presence of New York City Police Department (NYPD) personnel (including over 1,000 additional officers) and increased enforcement of transit rules of conduct. These initiatives have been praised by some New Yorkers for their efforts to increase subway security, but have also been criticized as not fully addressing underlying factors related to housing and mental health (Snyder 2023).

Various theoretical perspectives point to different mechanisms by which an increased police presence could influence perceptions of safety. Deterrence theory would posit that heightened police presence increases perceived risk of apprehension, thus deterring activities that could decrease perceptions of safety (Pratt et al. 2006). From an epidemiological perspective, increasing the number of police likely increases the number of police interactions with the public, potentially leading to higher arrest rates without necessarily leading to deterrence. Fundamental cause theory underscores how increases in police presence may not improve actual or perceived safety for all populations (Link and Phelan 1995; Clouston and Link 2021). This theory helps contextualize how increasing police presence can in fact exacerbate existing health inequities and further harm community health and social cohesion for marginalized communities that are already disproportionately subjected to more intensive policing and associated stress, disruption, and potential for violent encounters (Spolum et al. 2023; DeVylder et al. 2022).

Despite media and political focus on the issue of public safety on transit, there is little research that critically examines factors related to subway safety following the COVID-19 pandemic state of emergency declaration and initiatives that have been enacted in NYC. This study aimed to quantify the effects of the COVID-19 pandemic and NYC’s Subway Safety Plan on rates of complaints to and arrests by the NYPD Transit Bureau. We conducted interrupted time-series analyses of monthly complaints and arrests per one million riders on NYC subways from September 2018 to August 2023. We also examined trends by complaint and arrest type as well as changes in proportion of arrests by demographic and geographic groups. The findings of this analysis will help elucidate whether the COVID-19 pandemic state of emergency declaration had a measurable effect on changes to complaints and arrests within the NYC transit system and whether any changes were then influenced by the Subway Safety Plan.

## Methods

### Study setting and design

The setting for this interrupted time-series analysis was the NYC subway system from September 2018 to August 2023. The first case of COVID-19 was diagnosed in NYC on February 29, 2020 (COVID-19 Outbreak — New York City 2020), and on March 7, 2020 a state of emergency was declared to help the state of New York control the spread of the virus (At Novel Coronavirus Briefing, Governor Cuomo Declares State of Emergency to Contain Spread of Virus | Governor Kathy Hochul 2024). NYC’s mayor released the Subway Safety Plan on February 18, 2022 (The Official Website of the City of New York 2022). We utilized autoregressive integrated moving average (ARIMA) models applied to monthly time-series data to estimate the impact of the COVID-19 pandemic state of emergency declaration and the Subway Safety Plan on complaints to and arrests by the NYPD Transit Bureau.

### Data and variables

The independent measures in this analysis were the COVID-19 pandemic state of emergency declaration and the Subway Safety Plan announcement. Dummy variables were created for both interruptions, with the COVID-19 pandemic state of emergency declaration interruption variable coded as “0” for months prior to March 2020 and “1” after, and the Subway Safety Plan announcement interruption variable coded as “0” for months prior to February 2022 and “1” after. This step term (i.e., “0” pre-interruption and “1” post-interruption) was selected as opposed to a ramp term in which the interruption would be represented as a linear increase, as this approach fit most closely with the theory of a sudden shift (both in the case of the COVID-19 pandemic state of emergency declaration and the start of the Subway Safety Plan) and produced the best model fit statistics.

The dependent measures in this analysis were transit complaints, arrests, and arrest proportions, which we position as factors related to perceptions of safety. We accessed publicly available data on NYPD complaints and arrests and MTA subway ridership from the New York State and NYC open data portals (NYC Open Data 2024a; NYC Open Data 2024b; NYC Open Data 2024c; NYC Open Data 2024d; MTA Monthly Ridership/Traffic Data: Beginning 2008). Only complaints and arrests which were reported to the NYPD Transit Bureau, who are responsible for maintaining security on the NYC subway (Transit—NYPD 2024), were included in this analysis. We quantified incidence rates of complaints and arrests per one million subway riders per month and conducted additional subgroup analyses by complaint and arrest type. NYPD key codes were used to identify the five most common complaint and arrest types: assault, criminal mischief (e.g., intentional property damage such as graffiti (NYS Open Legislation | NYSenate.gov 2024a), grand larceny, harassment, and theft of services (i.e., fare evasion (Reports—Subway Fare Evasion—NYPD 2024; NYS Open Legislation | NYSenate.gov 2024b). Additionally, we examined trends in arrests by demographic and geographic groups over time. Because monthly ridership data were not available by demographic and geographic subgroup, we instead quantified the proportion of total arrests over time for each of the following variables: arrest borough and reported race, sex, and age group of the arrested individual. Due to low sample size, results for the following demographic and geographic groups, each of which represented less than 1% of arrests, are not presented: American Indian/Alaska Native, Sex Unknown, Age 65+, and Staten Island.

### Statistical analysis

We conducted interrupted time-series analyses using ARIMA models to estimate changes in transit complaints, arrests, and arrest proportions before and after the COVID-19 pandemic state of emergency declaration and the Subway Safety Plan announcement. While several methods are available for interrupted time-series analysis, ARIMA models are useful tools for analyzing time-series data with non-linear trends in which population-level interruptions or interventions are present (Schaffer et al. 2021). ARIMA models are expressed as (*p, d, q*) where *p* represents the order of the autoregressive portion of the model, *d* represents the degree of differencing in the model, and *q* represents the order of the moving average component of the model, each of which helps to control for underlying trends, seasonality, and autocorrelation. Seasonal ARIMA models are expressed as (*p*, *d*, *q*) × (*P*, *D*, *Q*), where *P*, *D*, and *Q* represent the seasonal autoregressive, differencing, and moving average components (Schaffer et al. 2021). We utilized the auto.arima function in the R forecast package (Hyndman and Khandakar 2008; R Core Team 2023) to automate the model selection process, which chooses the best fitting model by optimizing the Akaike Information Criterion (AIC) or Bayesian Information Criterion (BIC). To ensure appropriate model selection prior to adding our interruption variables, we conducted Ljung-Box tests for white noise (Burns 2002), examined the autocorrelation function (ACF) and partial autocorrelation function (PACF) plots, and evaluated the significance of the autoregressive and moving average coefficients. Additionally, we compared lower order models with those selected by the auto.arima function to identify the most parsimonious model while still maintaining good fit.

After identifying the most appropriate ARIMA model for each of our outcomes of interest, we tested the association between our COVID-19 and Subway Safety Plan interruption variables and transit complaints, arrests, and arrest proportions. Both the COVID-19 pandemic state of emergency declaration and Subway Safety Plan announcement interruptions were included in each model to control for the impact of the other interruption. Due to the large number of hypotheses tested for each interruption of interest, we implemented a Bonferroni correction to maintain an overall α of 0.05 (Dunn 1961). To create simultaneous 95% confidence intervals across the 27 tests for each interruption, each Bonferroni-corrected confidence interval presented was constructed using an α of 0.05 ÷ 27 = 0.0019.

## Results

**Fig. 1:**
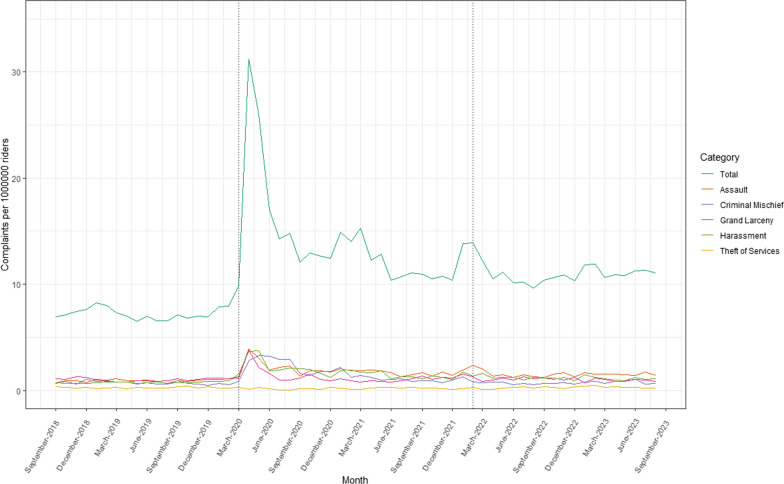
Complaints per 1 million riders to the NYPD Transit Bureau, September 2018–August 2023

**Fig. 2:**
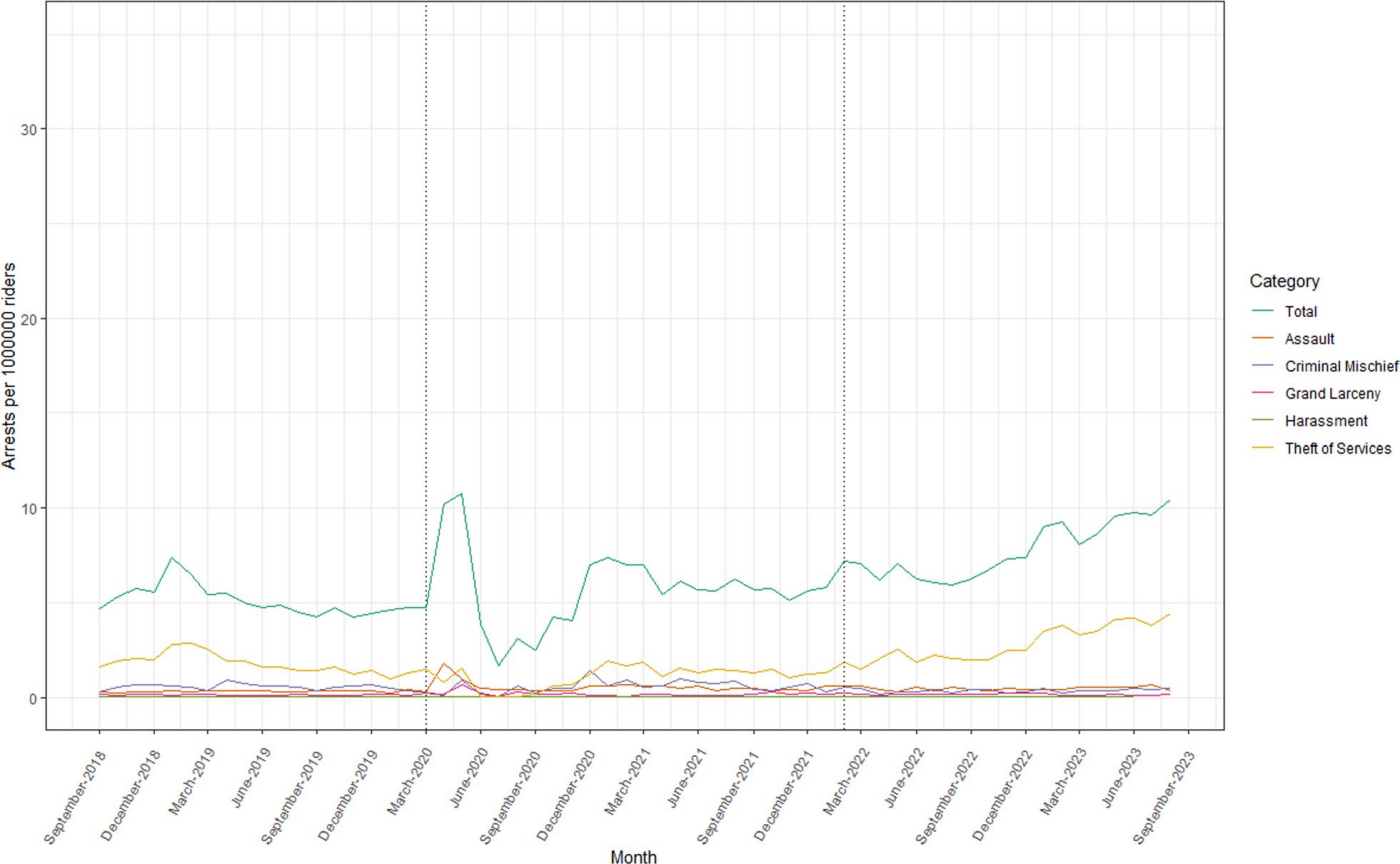
Arrests per 1 million riders by the NYPD Transit Bureau, September 2018–August 2023

**Fig. 3:**
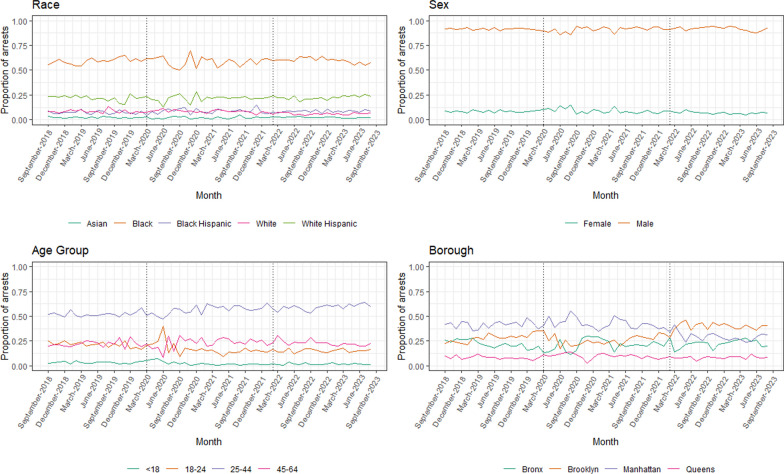
Proportion of NYPD Transit Bureau arrests by race, sex, age group, and borough

In the 60 months from September 2018 to August 2023, there were 51,929 unique complaints to and 32,898 unique arrests by the NYPD Transit Bureau. Before the COVID-19 pandemic state of emergency declaration in March 2020, there were an average of 7.22 complaints (SD = 0.52) and 5.13 arrests (SD = 0.81) per 1,000,000 subway riders each month. From March 2020 to January 2022, these numbers increased to 13.97 complaints (SD = 5.04) and 5.68 arrests (SD = 2.10) per 1,000,000 subway riders each month.. After the announcement of the Subway Safety Plan in February 2022, there were an average of 11.05 complaints (SD = 0.95) and 7.78 arrests (SD = 1.47) per 1,000,000 subway riders each month. The most common complaint category was assault, while the most common arrest category was theft of services. Individuals racialized as Black and identified as male and between the ages of 25 to 44 represented the largest proportions of people arrested. Between September 2018 and January 2022, most transit arrests occurred in Manhattan, while after the Subway Safety Plan announcement, the majority occurred in Brooklyn. Monthly summary statistics of complaints, arrests, and arrest proportions can be found in Table 1. Trends over time can be viewed in Figs. 1, 2 and 3.

**Table 1:** Monthly summary statistics of NYPD Transit Bureau complaints and arrests

Variable	Sept 2018–Feb 2020	Mar 2020–Jan 2022	Feb 2022–Aug 2023
	Mean (SD)	Mean (SD)	Mean (SD)
Complaints per 1,000,000 riders per month	7.223 (0.522)	13.971 (5.042)	11.049 (0.953)
Assault	0.918 (0.130)	1.887 (0.588)	1.541 (0.275)
Criminal Mischief	0.713 (0.149)	1.627 (0.836)	0.728 (0.126)
Grand Larceny	1.008 (0.159)	1.266 (0.642)	1.037 (0.164)
Harassment	0.754 (0.078)	1.769 (0.688)	1.173 (0.185)
Theft of Services	0.261 (0.065)	0.173 (0.092)	0.279 (0.093)
Arrests per 1,000,000 riders per month	5.133 (0.812)	5.677 (2.096)	7.775 (1.465)
Assault	0.317 (0.050)	0.547 (0.310)	0.469 (0.098)
Criminal Mischief	0.557 (0.157)	0.563 (0.327)	0.364 (0.110)
Grand Larceny	0.120 (0.029)	0.177 (0.122)	0.157 (0.043)
Harassment	0.003 (0.004)	0.000 (0.000)	0.004 (0.007)
Theft of Services	1.789 (0.517)	1.090 (0.577)	2.814 (0.947)
Proportion of arrests by race			
Asian	0.023 (0.006)	0.020 (0.011)	0.023 (0.006)
Black	0.592 (0.030)	0.587 (0.049)	0.599 (0.025)
Black Hispanic	0.075 (0.016)	0.087 (0.025)	0.085 (0.012)
White	0.085 (0.018)	0.085 (0.015)	0.061 (0.011)
White Hispanic	0.219 (0.028)	0.216 (0.033)	0.224 (0.019)
Proportion of arrests by sex			
Female	0.084 (0.009)	0.089 (0.026)	0.070 (0.012)
Male	0.916 (0.009)	0.911 (0.026)	0.919 (0.020)
Proportion of arrests by age			
< 18	0.033 (0.010)	0.024 (0.019)	0.017 (0.009)
18–24	0.215 (0.028)	0.170 (0.063)	0.153 (0.017)
25–44	0.522 (0.026)	0.564 (0.044)	0.591 (0.030)
45–64	0.221 (0.033)	0.232 (0.052)	0.229 (0.028)
Proportion of arrests by borough			
Bronx	0.221 (0.036)	0.211 (0.057)	0.225 (0.040)
Brooklyn	0.280 (0.040)	0.260 (0.047)	0.395 (0.039)
Manhattan	0.415 (0.036)	0.425 (0.052)	0.295 (0.042)
Queens	0.081 (0.017)	0.098 (0.024)	0.083 (0.014)

We specified ARIMA models for complaints per 1,000,000 riders per month and arrests per 1,000,000 riders per month, with subgroup analyses by complaint and arrest category. Additional models were specified for proportion of arrests by demographic and geographic groups. Model parameters and fit statistics can be found in Additional file 1: Supplementary Table 1.

Table 2 presents ARIMA model results for the effects of both the COVID-19 pandemic state of emergency declaration and the Subway Safety Plan announcement on transit complaints and arrests. After the COVID-19 pandemic state of emergency declaration, changes were observed in rates of complaints to the NYPD Transit Bureau. There was an 84% increase (i.e., an absolute increase of 6.07 per 1,000,000 riders, CI 1.42, 10.71) in complaints to the NYPD Transit Bureau, including a 99% increase (0.91 per 1,000,000 riders, CI 0.42, 1.41) in complaints for assault and a 125% increase in complaints for harassment (0.94 per 1,000,000 riders, CI 0.29, 1.60). Following the Subway Safety Plan announcement, an increase of 0.004 arrests per 1,000,000 riders (CI 0.001, 0.007) was observed for arrests for harassment. We also observed changes in the composition of the population of individuals arrested, including decreases in the proportion of arrests for individuals racialized as White (− 0.02, CI − 0.04, − 0.01) and proportion of arrests in the borough of Manhattan (− 0.13, CI − 0.17, − 0.09).

**Table 2:** Results of ARIMA models examining impacts of COVID-19 pandemic and Subway Safety Plan on NYPD Transit Bureau complaints and arrests and arrest proportions

Model	COVID-19 pandemic	Subway Safety Plan
	Coefficient (CI)	Coefficient (CI)
Complaints per 1,000,000 riders per month	6.065 (1.420, 10.711)	− 2.359 (− 6.891, 2.173)
Assault	0.913 (0.419, 1.406)	− 0.309 (− 0.783, 0.166)
Criminal Mischief	0.578 (− 0.371, 1.527)	− 0.636 (− 1.546, 0.274)
Grand Larceny	0.240 (− 0.303, 0.783)	− 0.230 (− 0.764, 0.305)
Harassment	0.942 (0.287, 1.598)	− 0.536 (− 1.180, 0.107)
Theft of Services	− 0.080 (− 0.198, 0.038)	0.096 (− 0.021, 0.213)
Arrests per 1,000,000 riders per month	− 0.140 (− 3.402, 3.123)	1.278 (− 2.038, 4.594)
Assault	0.221 (− 0.004, 0.447)	− 0.074 (− 0.295, 0.146)
Criminal Mischief	− 0.107 (− 0.728, 0.514)	− 0.028 (− 0.805, 0.749)
Grand Larceny	0.056 (− 0.021, 0.134)	− 0.020 (− 0.096, 0.057)
Harassment	− 0.003 (− 0.006, 0.000)	0.004 (0.001, 0.007)
Theft of Services	0.066 (− 1.212, 1.344)	0.494 (− 0.781, 1.770)
Proportion of arrests by race		
Asian	− 0.003 (− 0.011, 0.005)	0.003 (− 0.004, 0.011)
Black	− 0.005 (− 0.041, 0.031)	0.013 (− 0.022, 0.048)
Black Hispanic	0.012 (− 0.007, 0.030)	− 0.002 (− 0.020, 0.016)
White	− 0.000 (− 0.014, 0.014)	− 0.024 (− 0.038, − 0.010)
White Hispanic	− 0.003 (− 0.030, 0.023)	0.009 (− 0.017, 0.035)
Proportion of arrests by sex		
Female	0.015 (− 0.018, 0.049)	− 0.004 (− 0.041, 0.033)
Male	− 0.005 (− 0.024, 0.015)	0.008 (− 0.011, 0.027)
Proportion of arrests by age		
< 18	0.016 (− 0.020, 0.052)	0.002 (− 0.029, 0.032)
18–24	0.026 (− 0.073, 0.124)	0.005 (− 0.080, 0.089)
25–44	− 0.026 (− 0.109, 0.057)	− 0.021 (− 0.095, 0.054)
45–64	0.008 (− 0.036, 0.052)	0.000 (− 0.043, 0.044)
Proportion of arrests by borough		
Bronx	− 0.023 (− 0.095, 0.048)	0.026 (− 0.044, 0.096)
Brooklyn	− 0.036 (− 0.138, 0.066)	0.045 (− 0.084, 0.174)
Manhattan	0.010 (− 0.032, 0.053)	− 0.130 (− 0.172, − 0.088)
Queens	0.017 (− 0.006, 0.040)	− 0.015 (− 0.037, 0.008)

## Discussion

Throughout our study period, an average 100,206 rides occurred on transit per month for each complaint reported to the NYPD Transit Bureau, suggesting that a typical subway ride poses minimal risk of a rider experiencing a behavior warranting a complaint to police. However, our interrupted time-series analysis found changes in patterns of both complaints to and arrests by police in the NYC subway system following the COVID-19 pandemic state of emergency declaration and the announcement of the Subway Safety Plan. We identified large increases in relative rates of complaints for assault and harassment during the pandemic period, aligning with news media reports of an uptick in subway-related crime (Zraick et al. 2022; New York City Choking Death Revives Debate Over Subway Crime | Reuters 2024; New York Subway Crime: What is Perception, What is Real, and How to Fix it | CNN 2024). Following the enactment of the Subway Safety Plan, decreases were not observed in rates of complaints to the NYPD Transit Bureau, though there was a small increase in the rate of arrests for harassment. This could suggest an increase in police presence leading to stronger enforcement of transit policies, though it is unclear why this effect is specific to harassment and not observed for any other type of arrest. We also observed changes in the demographic and geographic composition of the population of individuals arrested, including a small (2%) decrease in proportions of arrests for individuals racialized as White, as well as a larger decrease in the proportion of arrests that occurred in the borough of Manhattan (13%). Because there was not an absolute decrease in arrest rates during this period, this shift may suggest changes in the geographic distribution of transit policing throughout the city, rather than reductions in policing within Manhattan.

This is the first study to specifically examine trends in complaints to and arrests by the NYPD Transit Bureau within the NYC subway system following the COVID-19 pandemic state of emergency declaration. The findings of increased complaints to police during the COVID-19 pandemic are in line with previous research in NYC (Koppel et al. 2023). Additionally, the racial disparities in arrest proportions are similar to those found in previous research on policing within the transit system (Carter and Johnson 2023). When considering theories of crime and violence, we did not observe findings in line with deterrence theory, which would predict that increases in police presence would reduce complaints through higher perceived consequences. The lack of an observable decrease in complaints may be more in line with an epidemiological perspective, which would predict that complaint rates will increase due to greater police-public interactions (i.e., more police present to whom reports can be made), resulting in no perceived change in overall complaint rates.

The findings of this study have important implications for future approaches to maintaining health and safety in the transit environment. Despite the implementation of the Subway Safety Plan and substantial increases in police spending (Gothamist 2023), rates of complaints on the NYC subway remain elevated after the onset of the COVID-19 pandemic. Further, beyond the Subway Safety Plan alone, MTA and law enforcement officials have stated that they are pursuing an approach of strong fare enforcement, which they theorize will result in lower rates of more serious crime over time by preventing potential offenders from entering the subway system (Ley 2024). We see evidence of this broader strategy in the disparity between the most common complaint category (i.e., assault) and the most common arrest category (i.e., theft of services) observed in this study’s descriptive statistics. However, though it is possible that this approach may require more time to have the intended effect, our analysis did not identify measurable decreases in overall complaint or arrest rates on the subway for any complaint or arrest type following the Subway Safety Plan’s announcement.

Additionally, this strategy may have unintended consequences that are not captured in this study, warranting examination of additional or different approaches to addressing subway safety. While a larger police presence and stronger fare enforcement may increase perceptions of safety for some riders, for other individuals, particularly youth racialized as Black, police exposure can be associated with feelings of distress and fear (Jindal et al. 2022). Individuals racialized as Black and Hispanic are over-represented in fines, summons, and arrests for fare evasion (i.e., theft of services) to such an extent that in 2020 the New York Attorney General opened “an investigation into whether the New York City Police Department (NYPD) has been targeting communities of color through its enforcement of the ‘theft of services’ law and the Metropolitan Transit Authority’s (MTA) ‘fare evasion’ regulations” (Carter and Johnson 2023; Attorney General James Launches Investigation into NYPD For Alleged Targeting of Communities of Color on NYC Subways 2020). Such arrests for minor offenses such as fare evasion may unnecessarily involve individuals in the criminal legal system, creating lasting and cascading effects for their health and well-being (Doherty et al. 2022). Other approaches not known to be associated with harm that may improve perceptions of safety on transit include increased ridership through wait time reductions, increases in non-police subway staff, and expansion of reduced fare programs (Riders Alliance 2024). Additionally, improvements to station and train cleanliness and the physical transit environment could decrease the incidence of crime and violence, as similar interventions have in other settings (Garvin et al. 2013; Chalfin et al. 2022). Such changes not only have the potential to improve perceptions of safety within the subway system, but also the quality of the ridership experience.

### Limitations

This study has several limitations. First, the NYPD complaints and arrests data are only two indirect ways of assessing factors related to transit safety. It is not clear whether any increases in complaints or arrests represent an increase in behaviors that reduce safety or simply an increase in police presence within stations to whom these behaviors could be reported. Moreover, we cannot be certain that the key codes used for arrests and complaints reliably and accurately categorize the behaviors that occurred, creating the possibility for misclassification or missing data, which could bias our results. Second, while both the COVID-19 pandemic and the Subway Safety Plan were large changes for the subway system and life in NYC, these changes may not have been as abrupt as represented by the coding of the interruption variables. Future studies could use transfer functions to examine more complex impacts, such as gradual trends or pulses followed by decays (Schaffer et al. 2021). Third, and similarly, there were a number of co-occurring events, including protests for racial justice and changes in city demographics and commuting patterns that may have resulted in unmeasured confounding. Fourth, the lack of data on ridership by demographic and geographic group limited our ability to examine trends for these groups beyond arrest proportions over time. Finally, this analysis was restricted to the NYC subway system, meaning the findings may not be generalizable to other urban areas or other transit systems.

## Conclusions

Ensuring equitable access to public transit is an even more daunting task following the beginning of the COVID-19 pandemic, which has taken an enormous toll on the physical, mental, and financial health of communities worldwide. The consequences of this event have been borne out across urban landscapes throughout the globe and have had particularly negative effects on transit systems and the benefits they provide. This study identified large and sustained increases in rates of complaints to the NYPD Transit Bureau following the start of the COVID-19 pandemic in NYC, which do not appear to have been fully remedied by the Subway Safety Plan. We also observed changes in the demographic and geographic composition of the population of individuals arrested. Future research should examine other interventions designed to increase perceptions of safety implemented within the NYC subway, and in transit systems globally, for both effectiveness and equity.

### Supplementary Information

**Additional file1**. Model parameters and fit statistics for selected ARIMA models.

## Data Availability

All data used for this study are publicly available from NYC Open Data (https://opendata.cityofnewyork.us/) and the New York State data portal (NYC Open Data 2024a; NYC Open Data 2024b; NYC Open Data 2024c; NYC Open Data 2024d; MTA Monthly Ridership/Traffic Data: Beginning 2008).

## Abbreviations

ACF: Autocorrelation function
AIC: Akaike information criterion
ARIMA: Autoregressive integrated moving average
BIC: Bayesian information criterion
CI: Confidence interval
MTA: Metropolitan Transportation Authority
NYC: New York City
NYPD: New York City Police Department
PACF: Partial autocorrelation function
SD: Standard deviation

## References

[CR1] At Novel Coronavirus Briefing, Governor Cuomo Declares State of Emergency to Contain Spread of Virus | Governor Kathy Hochul. 2024 [cited 2024 Feb 2]. Available from: https://www.governor.ny.gov/news/novel-coronavirus-briefing-governor-cuomo-declares-state-emergency-contain-spread-virus.

[CR2] Attorney General James Launches Investigation into NYPD For Alleged Targeting of Communities of Color on NYC Subways. 2020 [cited 2024 Apr 3]. Available from: https://ag.ny.gov/press-release/2020/attorney-general-james-launches-investigation-nypd-alleged-targeting-communities.

[CR3] Böcker L, Olsson LE, Priya Uteng T, Friman M (2023). Pandemic impacts on public transport safety and stress perceptions in Nordic cities. Transp Res Part Transp Environ.

[CR4] Bosman J, Kasakove S, Cowan J, Fausset R. Cities want to return to prepandemic life. One obstacle: transit crime. The New York Times. 2022 [cited 2024 Feb 8]; Available from: https://www.nytimes.com/2022/04/25/us/public-transit-crime.html.

[CR5] Bureau UC. Census.gov. 2019 [cited 2024 Feb 7]. Commuting by Public Transportation in the United States: 2019. Available from: https://www.census.gov/library/publications/2021/acs/acs-48.html.

[CR6] Burns P. Robustness of the Ljung-Box Test and its Rank Equivalent. Rochester, NY. 2002 [cited 2024 Feb 6]. Available from: https://papers.ssrn.com/abstract=443560.

[CR7] Carter TJ, Johnson LT (2023). “Blacks can’t jump”: the racialization of transit police responses to fare evasion. Race Justice.

[CR8] Chalfin A, Hansen B, Lerner J, Parker L (2022). Reducing crime through environmental design: evidence from a randomized experiment of street lighting in New York City. J Quant Criminol.

[CR9] Clouston SAP, Link BG (2021). A retrospective on fundamental cause theory: state of the literature, and goals for the future. Annu Rev Sociol.

[CR10] COVID-19 Outbreak — New York City. PMC. 2020 [cited 2024 Feb 2]. Available from: https://www.ncbi.nlm.nih.gov/pmc/articles/PMC7676643/

[CR11] DeVylder JE, Anglin DM, Bowleg L, Fedina L, Link BG (2022). Police violence and public health. Annu Rev Clin Psychol.

[CR12] Doherty EE, Green KM, Ensminger ME (2022). Long-term consequences of criminal justice system intervention: the impact of young adult arrest on midlife health behaviors. Prev Sci.

[CR13] Dunn OJ (1961). Multiple comparisons among means. J Am Stat Assoc.

[CR14] Garvin EC, Cannuscio CC, Branas CC (2013). Greening vacant lots to reduce violent crime: a randomised controlled trial. Inj Prev J Int Soc Child Adolesc Inj Prev.

[CR15] Gothamist. NYPD overtime pay in the subway went from $4 million to $155 million this year. 2023 [cited 2024 Jan 22]. Available from: https://gothamist.com/news/nypd-overtime-pay-in-the-subway-went-from-4-million-to-155-million-this-year.

[CR16] Hyndman RJ, Khandakar Y (2008). Automatic time series forecasting: the forecast package for R. J Stat Softw.

[CR17] Jindal M, Mistry KB, Trent M, McRae A, Thornton RLJ (2022). Police exposures and the health and well-being of Black Youth in the US: a systematic review. JAMA Pediatr.

[CR18] Kaufman S, Moss ML, Tyndall J, Hernandez J. Mobility, economic opportunity and New York City neighborhoods. Rochester, NY 2014 [cited 2024 Feb 7]. Available from: https://papers.ssrn.com/abstract=2598566.

[CR19] Koppel S, Capellan JA, Sharp J (2023). Disentangling the impact of Covid-19: an interrupted time series analysis of crime in New York City. Am J Crim Justice.

[CR20] Ley A. The Challenge of Making New York’s 472 Subway Stations Safer. The New York Times. 2024 [cited 2024 Apr 1]. Available from: https://www.nytimes.com/2024/03/28/nyregion/subway-shoving-manhattan-safety.html.

[CR21] Li N, Kim YA (2023). Subway station and neighborhood crime: an egohood analysis using subway ridership and crime data in New York City. Crime Delinq.

[CR22] Link BG, Phelan J (1995). Social conditions as fundamental causes of disease. J Health Soc Behav.

[CR23] MTA. 2022 [cited 2024 Feb 1]. MTA Customers Count Spring 2022 Survey Results. Available from: https://new.mta.info/article/mta-customers-count-spring-2022-survey-results.

[CR24] MTA Monthly Ridership/Traffic Data: Beginning | State of New York. 2008 [cited 2024 Jan 22]. Available from: https://data.ny.gov/Transportation/MTA-Monthly-Ridership-Traffic-Data-Beginning-Janua/xfre-bxip/about_data.

[CR25] MTA. 2024 [cited 2024 Feb 6]. Riding the subway. Available from: https://new.mta.info/guides/riding-the-subway.

[CR26] New York City Choking Death Revives Debate Over Subway Crime | Reuters. 2024 [cited 2024 Feb 1]. Available from: https://www.reuters.com/world/us/new-york-city-choking-death-revives-debate-over-subway-crime-2023-05-09/.

[CR27] New York Subway Crime: What is Perception, What is Real, and How to Fix it | CNN. 2024 [cited 2024 Feb 1]. Available from: https://www.cnn.com/2022/10/23/us/new-york-subway-crime-adams-miller/index.html.

[CR28] NYPD Arrest Data (Year to Date) | NYC Open Data. 2024a [cited 2024 Feb 6]. Available from: https://data.cityofnewyork.us/Public-Safety/NYPD-Arrest-Data-Year-to-Date-/uip8-fykc/about_data.

[CR29] NYPD Arrests Data (Historic) | NYC Open Data. 2024b [cited 2024 Feb 6]. Available from: https://data.cityofnewyork.us/Public-Safety/NYPD-Arrests-Data-Historic-/8h9b-rp9u/about_data.

[CR30] NYPD Complaint Data Current (Year to Date) | NYC Open Data. 2024c [cited 2024 Feb 6]. Available from: https://data.cityofnewyork.us/Public-Safety/NYPD-Complaint-Data-Current-Year-To-Date-/5uac-w243/about_data.

[CR31] NYPD Complaint Data Historic | NYC Open Data. 2024d [cited 2024 Jan 23]. Available from: https://data.cityofnewyork.us/Public-Safety/NYPD-Complaint-Data-Historic/qgea-i56i/about_data.

[CR32] NYS Open Legislation | NYSenate.gov. 2024a. [cited 2024 Mar 28]. Available from: https://www.nysenate.gov/legislation/laws/PEN/145.00

[CR33] NYS Open Legislation | NYSenate.gov. 2024b [cited 2024 Apr 10] Available from: https://www.nysenate.gov/legislation/laws/PEN/165.15.

[CR34] Pratt TC, Cullen FT, Blevins KR, Daigle LE, Madensen TD (2006). The empirical status of deterrence theory: a meta-analysis. Taking stock: the status of criminological theory.

[CR35] Public Transportation in the US: A Driver of Health and Equity | Health Affairs Brief. 2024 [cited 2024 Feb 1]. Available from: https://www.healthaffairs.org/do/10.1377/hpb20210630.810356/full/

[CR36] Qi Y, Liu J, Tao T, Zhao Q (2023). Impacts of COVID-19 on public transit ridership. Int J Transp Sci Technol.

[CR37] R Core Team. R: A Language and Environment for Statistical Computing. Vienna, Austria: R Foundation for Statistical Computing. 2023. Available from: https://www.R-project.org/.

[CR38] Reports - Subway Fare Evasion - NYPD. 2024 [cited 2024 Apr 10] Available from: https://www.nyc.gov/site/nypd/stats/reports-analysis/subway-fare-evasion.page.

[CR39] Riders Alliance. Riders need a real safety plan for our subway. 2024 [cited 2024 Feb 6]. Available from: https://www.ridersalliance.org/news/riders-need-a-real-safety-plan-for-our-subways.

[CR40] Schaffer AL, Dobbins TA, Pearson SA (2021). Interrupted time series analysis using autoregressive integrated moving average (ARIMA) models: a guide for evaluating large-scale health interventions. BMC Med Res Methodol.

[CR41] Snyder L (2023). New York’s directive for mental health involuntary removals: the intersectional risk for unhoused New Yorkers with a serious mental illness. Columbia Soc Work Rev.

[CR42] Spolum MM, Lopez WD, Watkins DC, Fleming PJ (2023). Police violence: reducing the harms of policing through public health-informed alternative response programs. Am J Public Health.

[CR43] The official website of the City of New York. 2022 [cited 2024 Jan 22]. Mayor Adams Releases Subway Safety Plan, Says Safe Subway is Prerequisite for NYC’s Recovery. Available from: http://www.nyc.gov/office-of-the-mayor/news/087-22/mayor-adams-releases-subway-safety-plan-says-safe-subway-prerequisite-new-york-city-s.

[CR44] Transit - NYPD. 2024 [cited 2024 Mar 28]. Available from: https://www.nyc.gov/site/nypd/bureaus/transit-housing/transit.page.

[CR45] Wu Y, Ridgeway G (2021). Effect of public transit on crime: evidence from SEPTA strikes in Philadelphia. J Exp Criminol.

[CR46] Xiao C, Goryakin Y, Cecchini M (2019). Physical activity levels and new public transit: a systematic review and meta-analysis. Am J Prev Med.

[CR47] Zraick K, Kvetenadze T, Paris F. How safe is the subway? What those who work there have to say. The New York Times. 2022 [cited 2024 Jan 22]. Available from: https://www.nytimes.com/2022/11/04/nyregion/new-york-subway-safety.html.

