# Supplementary material for: Trends in police complaints and arrests on New York City subways, 2018 to 2023: an interrupted time-series analysis
Source: Inj Epidemiol. 2024 Apr 26;11:16. doi: 10.1186/s40621-024-00501-9 (PMC11055262; doi:10.1186/s40621-024-00501-9)
Supplement: Supplementary file 1 — Additional file1. Model parameters and fit statistics for selected ARIMA models. [file 40621_2024_501_MOESM1_ESM.docx]

**Supplementary Table 1. Model parameters and fit statistics**

| **Model** | **Parameters and fit** | **Q* statistic** | **Ljung-Box test p-value** |
| --- | --- | --- | --- |
| **Complaints per 1,000,000 riders per month** | (1,0,0) | 3.244 | 0.987 |
| Assault | (0,1,2) | 5.317 | 0.869 |
| Criminal Mischief | (1,0,0) | 11.134 | 0.432 |
| Grand Larceny | (1,0,0) | 3.437 | 0.984 |
| Harassment | (1,0,0) | 11.650 | 0.391 |
| Theft of Services | (2,0,0) | 11.411 | 0.326 |
| **Arrests per 1,000,000 riders per month** | (0,1,2) | 6.810 | 0.743 |
| Assault | (1,0,0) | 1.538 | 0.999 |
| Criminal Mischief | (2,0,2)x(1,0,0) | 9.002 | 0.253 |
| Grand Larceny | (0,0,0) | 9.576 | 0.653 |
| Harassment | (3,0,0) | 7.439 | 0.592 |
| Theft of Services | (1,1,0) | 8.958 | 0.626 |
| **Proportion of arrests by race** |  |  |  |
| Asian | (0,0,0) | 11.329 | 0.501 |
| Black | (0,0,0) | 8.643 | 0.733 |
| Black Hispanic | (0,0,0) | 10.399 | 0.581 |
| White | (0,1,1) | 15.762 | 0.150 |
| White Hispanic | (0,0,0) | 13.294 | 0.348 |
| **Proportion of arrests by sex** |  |  |  |
| Female | (0,1,1) | 10.570 | 0.480 |
| Male | (0,0,0) | 11.333 | 0.501 |
| **Proportion of arrests by age** |  |  |  |
| <18 | (1,1,1) | 5.461 | 0.858 |
| 18-24 | (0,1,1) | 14.110 | 0.227 |
| 25-44 | (0,1,1) | 10.745 | 0.465 |
| 45-64 | (2,0,0) | 11.621 | 0.311 |
| **Proportion of arrests by borough** |  |  |  |
| Bronx | (1,0,0) | 10.560 | 0.481 |
| Brooklyn | (0,1,1) | 8.742 | 0.646 |
| Manhattan | (0,1,1) | 12.476 | 0.329 |
| Queens | (1,0,0) | 4.134 | 0.966 |
